# Lytic activity by temperate phages of *Pseudomonas aeruginosa* in long-term cystic fibrosis chronic lung infections

**DOI:** 10.1038/ismej.2014.223

**Published:** 2014-12-02

**Authors:** Chloe E James, Emily V Davies, Joanne L Fothergill, Martin J Walshaw, Colin M Beale, Michael A Brockhurst, Craig Winstanley

**Affiliations:** 1 Institute of Infection and Global Health, University of Liverpool, Liverpool, UK; 2 School of Environment and Life Sciences, University of Salford, Manchester, UK; 3 Liverpool Heart and Chest Hospital, Liverpool, UK; 4 Department of Biology, University of York, York, UK

## Abstract

*Pseudomonas aeruginosa* is the most common bacterial pathogen infecting the lungs of cystic fibrosis (CF) patients. The transmissible Liverpool epidemic strain (LES) harbours multiple inducible prophages (LESϕ2; LESϕ3; LESϕ4; LESϕ5; and LESϕ6), some of which are known to confer a competitive advantage in an *in vivo* rat model of chronic lung infection. We used quantitative PCR (Q-PCR) to measure the density and dynamics of all five LES phages in the sputa of 10 LES-infected CF patients over a period of 2 years. In all patients, the densities of free-LES phages were positively correlated with the densities of *P. aeruginosa*, and total free-phage densities consistently exceeded bacterial host densities 10–100-fold. Further, we observed a negative correlation between the phage-to-bacterium ratio and bacterial density, suggesting a role for lysis by temperate phages in regulation of the bacterial population densities. In 9/10 patients, LESϕ2 and LESϕ4 were the most abundant free phages, which reflects the differential *in vitro* induction properties of the phages. These data indicate that temperate phages of *P. aeruginosa* retain lytic activity after prolonged periods of chronic infection in the CF lung, and suggest that temperate phage lysis may contribute to regulation of *P. aeruginosa* density *in vivo*.

## Introduction

Cystic fibrosis (CF) patients are subject to life-long chronic respiratory infections, most commonly with the bacterium *Pseudomonas aeruginosa*. Periodic exacerbation of symptoms occurs throughout the lifetime of CF patients leading to progressive deterioration of lung function. Phage particles have been detected in the sputa of CF patients (Ojeniyi *et al.,* 1991; Fothergill *et al.,* 2011). Metagenomic analysis of CF sputa has identified >450 viral genotypes, whereas most viruses were unknown, of those that could be identified as viruses the majority were infective against CF pathogens, including many *Pseudomonas* phages (Lim *et al.,* 2014). More generally, phages outnumber eukaryotic viruses both in abundance and diversity in the human virome (Reyes *et al.,* 2012) and are known to be present at various body sites including the gastrointestinal (Breitbart *et al.,* 2003; Kim *et al.,* 2011) and the respiratory tracts (Willner *et al.,* 2009). However, the *in vivo* ecological dynamics of temperate bacteriophages and their role during bacterial infections remain largely unknown.

Upon infection of a bacterial cell, a temperate phage can either complete the lytic cycle or integrate into the bacterial chromosome as a prophage, which may subsequently be induced to enter the lytic cycle by a range of bacterial or environmental cues (Little, 2005; Ghosh *et al.,* 2009). Because lysis is obviously detrimental to the individual host bacterium it is often assumed that integrated prophages will eventually lose their lytic activity, becoming cryptic. Whereas the selective forces and mechanisms driving this remain poorly understood, inactive prophage remnants have been detected in many bacterial species, and are thought to result from an ongoing process of phage decay (Brussow *et al.,* 2004). This could be owing to the accumulation of mutations; for example, to inactivate phage N-antiterminator genes (Desiere *et al.,* 2001) and portal protein genes (Lawrence *et al.,* 2001), preventing completion of the replicative cycle. By contrast, lytic activity may be retained if it enhances bacterial population fitness, for example, by acting as an anticompetitor strategy or through the release of virulence-related toxins upon lysis (Brussow *et al.,* 2004; Brown *et al.,* 2006; Willner *et al.,* 2009). Over longer evolutionary timescales, the domestication of prophages is thought to be an important process in the evolution of bacteria, leading to the origin of a number of phage-derived traits (for example, bacteriocins, killer particles etc.(Bobay *et al.,* 2014)).

The *P. aeruginosa* Liverpool epidemic strain (LES) exhibits increased antibiotic resistance levels compared with other *P. aeruginosa* isolates from CF patients (Ashish *et al.,* 2012) and is widespread across the UK (Martin *et al.,* 2013). Patients infected with this strain have been shown to suffer greater morbidity than those infected with other strains (Al-Aloul *et al.,* 2004; Fothergill *et al.,* 2012). The *P. aeruginosa* LESB58 genome contains five inducible prophages and transposon mutagenesis of this isolate identified several mutations in prophages encoding LESϕ2, LESϕ3 and LESϕ5 that reduced bacterial competitiveness in a rat model of chronic lung infection (Winstanley *et al.,* 2009), suggesting that the phages have a key role in the infection process. We have previously characterised the infection properties of several LES phages *in vitro*. Induction experiments demonstrated that free LESϕ2 was produced more rapidly and in higher numbers than LESϕ3 and LESϕ4 in response to norfloxacin. Each phage was shown to exhibit a different immunity profile and was able to infect a range of susceptible *P. aeruginosa* hosts (James *et al.,* 2012). Owing to a lack of suitable acceptor strains, we have thus far been unable to isolate and purify LESϕ5 and LESϕ6.

In this study, we used culture-independent quantitative PCR (Q-PCR) to follow the ecological dynamics of all 5 active LES phage populations in 188 expectorated sputum samples from 10 long-term LES-infected patients over a period of 28 months. To our knowledge, this represents the first longitudinal study of a bacterial pathogen and its temperate phages in a human chronic infection.

## Materials and methods

### Patients and Samples

Sputum samples (*n*=188) were collected from 10 LES-infected CF patients for diagnostic purposes over a period of >2 years (January 2009 to May 2011). The details of each patient and the sampling rationale have been described previously (Fothergill *et al.,* 2010; Mowat *et al.,* 2011). All patients had long-term LES infections (duration at beginning of the study ranged from >5 to >10 years). Table 1 and Supplementary Figure S1 describe the number and dates of acquired samples from each patient that were analysed for density of LES bacteria and LES phages. During routine visits 98 samples were collected when each patient was well (stable) and 90 samples were collected during periods of acute exacerbated symptoms of respiratory infection (acute). Sputa obtained during exacerbation periods included samples taken before and during aggressive intravenous antibiotic treatment (Table 1, Supplementary Figure S1). The criteria for diagnosing exacerbations were physician-based and have been described previously (Mowat *et al.,* 2011). Briefly, patients were considered to be undergoing an exacerbation if they showed signs of reduced lung capacity, increased sputum production and discoloration, increased temperature, cough, dyspnoea and malaise (Goss and Burns, 2007). Where known, antibiotics administered to patients during the study period are listed in Table 1. However, detailed information of the antibiotics administered during each exacerbation is very incomplete. Thus we were unable to fully assess the effect of different antibiotics on phage induction *in vivo*. This study was approved by the local Research Ethics Committee (REC reference 08/H1006/47).

### Detection of viable phage particles by plaque assay

We were unable to accurately determine phage densities by culture-dependent techniques for several reasons: (a) we lacked acceptor strains for LESϕ5 and LESϕ6; (b) culturable phages were indistinguishable by plaque morphology; and (c) sputum samples were routinely frozen upon collection, which reduced phage viability. However, to confirm the presence of viable phage particles in sputum samples, we quantified the density of culturable phages in 10 sputum samples from 3 LES-infected patients (CF3, CF4 and CF7). Sputum samples (50 μl) were treated with sputasol (200 μl) and incubated at 37 °C for 1 h, with shaking at 200 r.p.m. Treated samples were diluted with sterile phosphate-buffered saline. A rifampicin-resistant mutant (PAO1-rif) was created by successive passage in increasing rifampicin concentrations (method described by James *et al.,* 2001). This enabled enumeration of active phages capable of infecting PAO1 directly from unfiltered sputum. Briefly, mid-exponential phase PAO1-rif (OD_600_ 0.5; 100 μl) was added as an indicator host to treated sputum samples (400 μl). Rifampin (300 mg ml^−1^) was incorporated in the soft agar overlay (5 ml; 0.4% (w/v) Luria broth (LB) agar) to select for the indicator host and incubated overnight at 37 °C before the plaques were counted. This method only provides confirmation that active *P. aeruginosa* phages are present in the sputa. It does not accurately reflect abundance and does not discriminate between phages.

### Real-time Q-PCR

To overcome the limitations of culture-dependent methods, we have developed and validated a simple Q-PCR protocol to measure the density of each individual LES phage (James *et al.,* 2012). Each sputum sample was treated with an equal volume of Sputasol (Oxoid, Basingstoke, UK) and incubated at 37 ^o^C for 30 min, with shaking at 200 r.p.m. DNA was prepared from each treated sputum sample (400 μl) using the ‘Qiasymphony Virus/Pathogen DNA extraction kit' (Qiagen, Valencia, CA, USA) and the automated QIAsymphony machine (QIAGEN; pathogen complex 200 protocol). The protocol yielded 0.3–0.9 μg μl^−1^ DNA. Each sample was diluted 1:100 with sterile distilled H_2_O. The number of DNA copies of each LES phage in sputum and bacterial culture samples was quantified from extracted DNA. The number of specific copies detected for each phage was compared with a concentration gradient of known standards (James *et al.,* 2012). For each LES phage (LESϕ2–LESϕ6), two specific primer sets were used to quantify i) prophage and ii) total copies (10 primer sets in total). Differentiation between total phage and prophage copies, allowed free-phage densities to be calculated as the difference between these values as previously described (James *et al.,* 2012). Bacterial host density was quantified using primers specific for *P. aeruginosa* (PS21-6F1/PS21-6R1 and gyrPA-F1/gyrPA-R1) (Fothergill *et al.,* 2013). All primer sequences and targets are listed in Supplementary Table S1.

Q-PCR reactions (25 μl) contained 1 μM each primer pair and 1X Rotorgene-SYBR green super-mix (Qiagen). All primer sets were used with the same cycling conditions: 95 °C for 10 min; followed by 40 cycles of 95 °C for 10 s, 60 °C for 15 s and 72 °C for 20 s. Phage DNA copy numbers were quantified from DNA samples (1 μl) in triplicate using a Rotorgene cycler (Qiagen). Q-PCR data were analysed using Rotorgene Q series software 1.7 (Qiagen).

### Sputasol induction experiments

To test for any potential induction properties of sputasol, DNA was extracted from LESB58 cultures grown to mid-exponential phase in LB (James *et al.,* 2012) treated (in triplicate) with an equal volume of sputasol or LB for 30 min. DNA was prepared from each culture using a DNA mini kit (Qiagen) and phage densities estimated by Q-PCR as described for the sputum samples. No effect of sputasol on phage induction was observed (Supplementary Figure S2).

### Statistical analysis

To model phage densities (or phage-to-bacterium ratios), we fitted linear mixed effects models with maximum likelihood using the R package nlme (Pinheiro and Bates 2000) with and without temporal autocorrelated errors (an ARMA(1) model). Models with temporal autocorrelated errors were significant improvements over those without, and therefore we present only these models below. We included a random effect for patient ID and fixed effects for time, exacerbation, bacterial load and the interaction between bacterial load and exacerbation. We compared full models with and without temporally autocorrelated errors using a likelihood ratio test, and then used a backwards stepwise process to remove nonsignificant fixed effects until the minimum adequate model was identified. Models analysing normalised variables gave similar results to those analysing non-normalised data and are presented in the supplementary information (Supplementary Table S2a).

## Results

### Dynamics of total free-phage abundance

**Figure 1:**
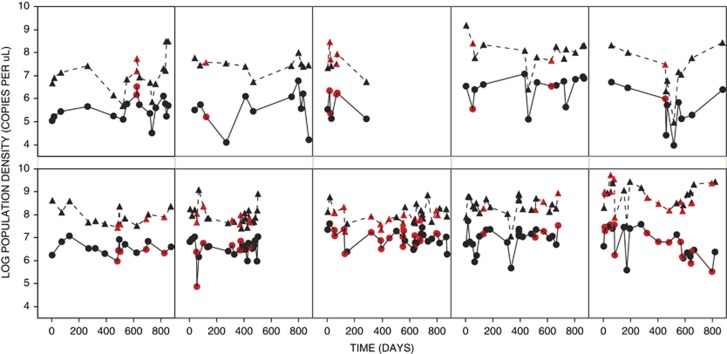
Longitudinal dynamics of total free-phage density and *P. aeruginosa* density in 10 CF patients. Q-PCR assays were used to enumerate free-LES phage (dotted line) and *P. aeruginosa* (solid line) densities from the sputa of 10 LES-infected CF patients (CF1–CF5 left to right top row and CF6–CF10 left to right bottom row) over a 2-year period. Samples were obtained from patients both during stable periods (black symbols) and during exacerbation of symptoms (red symbols). The dotted line represents the mean values of all free-LES phages (2,3,4,5 and 6) for each patient. The density of free-phage copies of each LES phage was calculated by subtracting prophage copies from total phage copies in each case.

**Figure 2:**
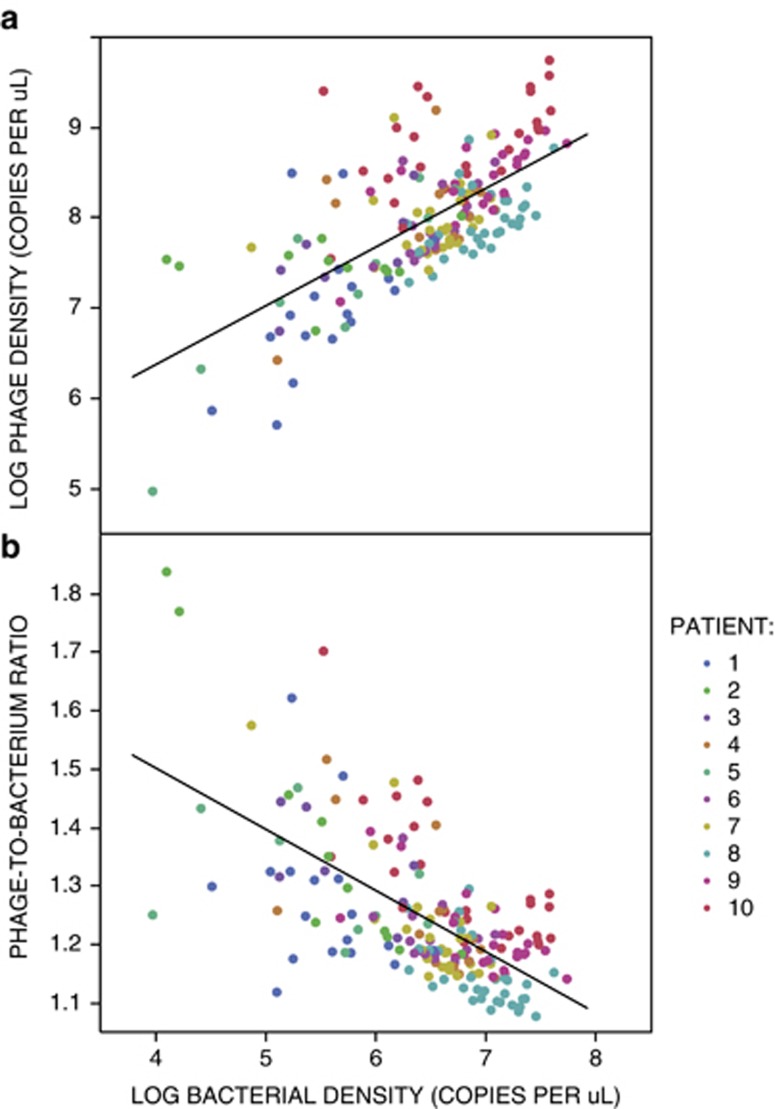
Relationships of phage density and phage-to-bacterium ratio with bacterial density. Data points represent sputum samples; patient identity is indicated by colour (see visual key for details); regression lines indicate significant relationships between variables. (**a**) The positive relationship between log10 phage density and log10 bacterial density; (**b**) The negative relationship between phage-to-bacterium ratio and log10 bacterial density.

**Figure 3:**
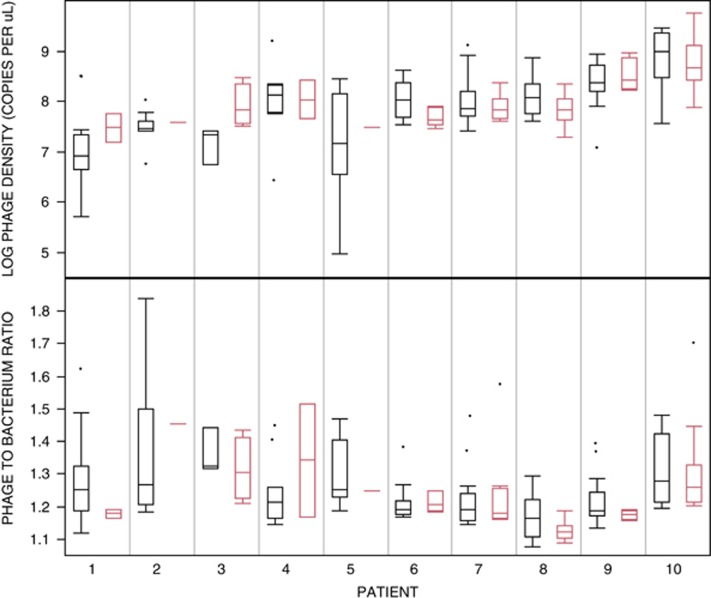
Phage density and phage-to-bacterium ratio are not affected by exacerbations. Outlier box-plots display phage density (upper panel) or phage-to-bacterium ratio (lower panel) in sputa from patients during stable periods (black) and exacerbations (red).

Plaque assays confirmed the presence of viable particles of the subset of culturable phages in all sputum samples that were tested (range 1.25 × 10^2^–1.39 × 10^4^ PFU ml^−1^). Free-LES phage DNA was detected in all patient sputa (9.35 × 10^4^–5.54 × 10^9^ copies μl^−1^) and, in general, total free-phage density (that is, the sum of all the free-LES phages present in the sample) exceeded that of *P. aeruginosa* within each sample (mean range 11-fold to 90-fold) (Figure 1 and Supplementary Table S3). We observed a positive linear relationship between total free-phage density and bacterial density (Figure 2a and Supplementary Table S2b; bacterial coefficient 0.607 ± 0.054, LRT=85.9, d.f.=1, *P*<0.001), consistent with ongoing lytic activity by the temperate phages, but no effect of time or exacerbations on total free-phage densities. Further, we observed a negative linear relationship between the phage-to-bacterium ratio and bacterial density (Figure 2b and Supplementary Table S2c bacterial coefficient -3.206± 0.484, LRT=108.4, d.f.=1, *P*<0.001), suggesting a role for phage lysis in regulation of bacterial densities. Time and exacerbations had no significant effect on phage-to-bacterium ratios. It is perhaps surprising that exacerbations were not associated with either a change in phage densities or a change in the phage-to-bacterium ratio (Figure 3), given that these episodes are associated with the administration of high-dose intravenous antibiotics. However, it should be noted that these patients were all subject to variable cocktails of antibiotics over several years irrespective of exacerbations (Table 1). Moreover, clinical data on antibiotic use in these patients was too incomplete to be used in analyses, and therefore effects of particular antibiotics on phage dynamics may have been missed.

### Abundance heirarchy among individual phages within lungs

**Figure 4:**
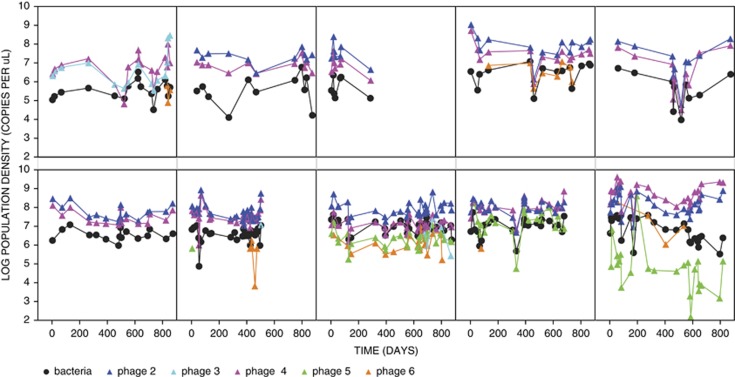
Densities of individual LES phage types in patient sputa exhibit hierarchical trends. The free-phage densities, calculated for each individual LES phage in the 10 CF patients analysed (CF1–CF5 left to right top row, and CF6–CF10 left to right bottom row). Each line represents one LES phage type; LESϕ2 (blue); LESϕ3 (cyan); LES ϕ4 (pink); LES ϕ5 (green); LES ϕ6 (orange); and *P. aeruginosa* (black circles). All Q-PCR assays were performed in triplicate and mean values are presented. The density of free-phage copies of each LES phage was calculated by subtracting prophage copies from total phage copies in each case.

Next, we considered each phage individually, observing a general hierarchy of free-phage densities, though the precise patterns were clearly influenced by the fact that the LES populations for each patient did not all share the same prophage complement (Table 1). Figure 4 illustrates the free-phage densities of individual LES phages for each of the patients. In the majority of patients (CF2–CF9) similar free-phage dynamics were observed, in that the density of free LESϕ2 was consistently higher than that of the other LES phages, closely followed by LESϕ4 (Figure 4). A positive correlation was observed between LESϕ2 and LESϕ4 densities in these patients (Supplementary Table S4). The dynamics observed in samples from patient CF10 (Figure 3) exhibited a change in the hierarchy of free phage, with considerably higher free-LESϕ4 densities observed. Despite consistent carriage of LES prophage 3, very little free LESϕ3 was detected in patients CF2–CF10. However, higher levels of free LESϕ3 (3.29 × 10^7^ copies μl^−1^) were observed in all sputa from patient CF1 (Figure 4), whose *P. aeruginosa* were the only populations not to carry prophage 2 (Table 1). We showed previously that LES populations exhibit genotypic diversity, including variation in carriage of LES prophages. In particular, the carriage of LES prophage 5 was not consistent in all individuals of a given LES population (Fothergill, *et al.*, 2010). In this study, prophage 5 was intermittently detectable in the sputum from patients 7 (up to 10^5^ copies μl^−1^) and 10 (10^2^–10^4^ copies μl^−1^). This explains the low density of free LESϕ5 in these patients. Free copies of LESϕ6 were not detected in the majority of sputum samples. Where free copies were detected, the density was lower than the host bacterial load (6.7 × 10^3^–1 × 10^7^ copies μl^−1^).

## Discussion

The levels of free-LES phages detected in all patients throughout this study suggest an active lytic cycle that may be promoted by the presence of H_2_O_2_ or DNA-damaging antibiotics in the CF lung (McGrath *et al.,* 1999; Fothergill *et al.,* 2011). Surprisingly, we observed no effect of patient exacerbation on total free-phage density, although this is consistent with previous studies showing that neither fluctuations in *P. aeruginosa* populations (Mowat *et al.,* 2011), nor in the wider bacterial population (Fodor *et al.,* 2012), show any relationship with the exacerbation period in chronically infected patients, despite the use of high-level intravenous antibiotic therapy. It is known that particular antibiotics can induce phage lysis and it is possible that different antibiotics regimes may have influenced differential induction of phages between patients. Indeed, we have shown previously that LES induction varies in response to different antibiotics (Fothergill *et al.,* 2011). Unfortunately, because records of antibiotic treatments for these patients were very incomplete, we were unable to explicitly test for effects of particular antibiotics in this study. This would in any case be difficult because of the extensive and varied use of antibiotics in this group of patients (Table 1), which was not restricted to periods of exacerbation.

Our data do however suggest that ongoing phage lysis may have a role in regulating bacterial density in the CF lung. Treatments that induced the lytic cycle of temperate phages could therefore offer a promising alternative or addition to standard antibiotic therapies, which in themselves often do not successfully reduce *P. aeruginosa* densities in long-term chronically infected patients (Foweraker, 2009; Mowat *et al.,* 2011). Several studies have demonstrated effective phage-antibiotic synergism in the reduction of bacterial numbers *in vitro* and *in vivo* (Hagens *et al.,* 2006; Comeau *et al.,* 2007; Knezevic *et al.,* 2013). However, this strategy would need to be considered with caution. Antibiotic therapies that induce stx phages of Shiga-toxigenic *E. coli* have been shown to increase expression of Shiga toxin genes that are encoded in the late region of the phage genome and thus increase cytotoxic damage and exacerbate symptoms (Matsushiro *et al.,* 1999). Although we have not identified any obvious virulence factors encoded in the late gene region of the LES phages (Winstanley, *et al.*, 2009; James, *et al.*, 2012), we cannot ignore the possibility that the lytic cycle might induce upregulation of virulence genes.

We demonstrate here that LESϕ2 was the most abundant free phage in 9-out-of-10 LES-infected patients. The hierarchy of free-LES phage in patient sputa was also observed in our previous studies of LES phage induction in *in vitro* bacterial cultures (James *et al.,* 2012). This suggests therefore that LESϕ2 is generally more readily induced or exhibits a more efficient lytic cycle than the other phages both *in vitro* and *in vivo*. In the sputa of patient CF1, who was infected by a LES that lacked prophage 2, LESϕ3 reached far higher abundances than observed in other patients, suggesting potential suppression of LESϕ3 lysis by LESϕ2 *in vivo*. In accordance with our previous *in vitro* observations of coinduction of lysis by prophages, we observed a degree of synchronisation of free-phage dynamics *in vivo*, suggesting that the phages may be responding to shared signals, which could include a wide variety of human host, bacterial and environmental triggers (Little, 2005). It is exceptionally difficult to disentangle to drivers of microbial dynamics *in vivo* due to the complexity of host microenvironments; future studies using laboratory models of the infection environment allowing the constituent drivers to be decomposed will be necessary to elucidate this (Wright *et al.,* 2013; Fothergill *et al.,* 2014).

The long-term maintenance of intact, active temperate phages in the LES genome despite substantial cell lysis suggests some selective or competitive advantage *in vivo*, consistent with previous work highlighting a loss of competitiveness observed following the introduction of mutations to some LES prophage regions (Winstanley *et al.,* 2009). One possibility is that free-phage particles produced by a subpopulation of LES could kill competing bacteria (Brown *et al.,* 2006). Indeed, LESϕ2, LESϕ3 and LESϕ4 are capable of infecting and lysing other clinical *P. aeruginosa* isolates (James *et al.,* 2012). Thus frequent induction of the lytic cycle may enhance the competitive ability of LES by promoting superinfection, which has been observed clinically (McCallum *et al.,* 2001), and preventing invasion of the lung by other strains of *P. aeruginosa*. Alternatively the prophages may contain accessory genes that contribute directly to LES fitness in the CF lung, which are only expressed during the lytic cycle, as observed for other pathogens (Wagner *et al.,* 2001).

Little is known about the consequences for the human host of the presence of large numbers of phage in the lung. However, high titre phage preparations have recently been found to interact with the immune system *in vivo* (Letkiewicz *et al.,* 2010). It has also been suggested that, following adherence to mucous, some phages may act as a form of innate host immunity enhancing host defences against bacterial pathogens (Barr *et al.,* 2013). Our findings of high free-phage abundances in CF lungs highlight the urgent need for research into the interaction of phages with host immunity, particularly in CF where dysfunctional immune responses contribute to pathological processes.

## Figures and Tables

**Table 1:** Patient LES phage complement and sputum sample summary

*Patient*	*ϕ Complement*	*Total*	*Stable*	*Exacerbation*	*Antibiotics*
CF1	3, 4, 6	17	14	3	TOB (N), AZT (O)
CF2	2, 3, 4, 6	11	9	2	CEPH (O), CEF (IV), COL (IV)
CF3^a^	2, 3, 4, 6	7	1	6	AZT (O), COL (N), CEF (IV), COL (IV)
CF4	2, 3, 4, 6	14	10	4	CEF (IV), COL (IV)
CF5	2, 3, 4, 6	10	7	3	
CF6	2, 3, 4, 6	16	9	7	AZT (O), COL (N)
CF7^a^	2, 3, 4, 5, 6	28	11	17	AZT (O), COL (N), CEF (IV), COL (IV), MER (IV), FOS (IV)
CF8	2, 3, 4, 5, 6	33	8	25	CEPH (O), FOS (IV), MER (IV), CEF (IV)
CF9	2, 3, 4, 5, 6	25	19	6	AZT (O), COL (N), TOB (N), MER (IV), COL (IV)
CF10	2, 3, 4, 5, 6	27	10	17	MER (IV), COL (IV)
Total		188	98	90	

Abbreviations: AZT, azithromycin; CF, cystic fibrosis; CEPH, cephadrine; CEF, ceftazidime; COL, colomycin; FOS, fosfomycin; IV, intravenous (used during exacerbations only); LES, Liverpool epidemic strain; MER, meropenem; N, nebulised; O, oral; TOB, tobramycin.

CF patient sputum contained LES variants that harboured different phage complements. Stable samples were collected during periods of relative patient health. Exacerbation samples were collected during periods of reduced lung function and hospitalisation of patients, who underwent antibiotic treatment for which data are incomplete. Antibiotics used during the period of the study are shown (where known): Route of administration is indicated in brackets.

a Patients CF3 and CF7 died before completion of this study.

## Notes

The authors declare no conflict of intrest.

## References

[bib1] Al-Aloul M Crawley J Winstanley C Hart CA Ledson MJ Walshaw MJ 2004 Increased morbidity associated with chronic infection by an epidemic *Pseudomonas aeruginosa* strain in CF patients Thorax 59 334 336 15047956 10.1136/thx.2003.014258 PMC1763796

[bib2] Ashish A Shaw M Winstanley C Ledson MJ Walshaw MJ 2012 Increasing resistance of the Liverpool epidemic strain (LES) of *Pseudomonas aeruginosa* (Psa) to antibiotics in cystic fibrosis (CF)—a cause for concern J Cyst Fibros 11 173 179 22146482 10.1016/j.jcf.2011.11.004

[bib3] Barr JJ Auro R Furlan M Whiteson KL Erb ML Pogliano J 2013 Bacteriophage adhering to mucus provide a non-host-derived immunity Proc Natl Acad Sci USA 110 10771 10776 23690590 10.1073/pnas.1305923110 PMC3696810

[bib4] Bobay LM Touchon M Rocha EP 2014 Pervasive domestication of defective prophages by bacteria Proc Natl Acad Sci USA 111 12127 12132 25092302 10.1073/pnas.1405336111 PMC4143005

[bib5] Breitbart M Hewson I Felts B Mahaffy JM Nulton J Salamon P 2003 Metagenomic analyses of an uncultured viral community from human feces J Bacteriol 185 6220 6223 14526037 10.1128/JB.185.20.6220-6223.2003 PMC225035

[bib6] Brown SP Le Chat L De Paepe M Taddei F 2006 Ecology of microbial invasions: amplification allows virus carriers to invade more rapidly when rare Curr Biol 16 2048 2052 17055985 10.1016/j.cub.2006.08.089

[bib7] Brussow H Canchaya C Hardt WD 2004 Phages and the evolution of bacterial pathogens: from genomic rearrangements to lysogenic conversion Microbiol Mol Biol Rev 68 560 602 15353570 10.1128/MMBR.68.3.560-602.2004 PMC515249

[bib8] Comeau AM Tetart F Trojet SN Prere MF Krisch HM 2007 Phage-Antibiotic Synergy (PAS): beta-lactam and quinolone antibiotics stimulate virulent phage growth PLoS One 2 e799 17726529 10.1371/journal.pone.0000799 PMC1949050

[bib9] Desiere F McShan WM van Sinderen D Ferretti JJ Brussow H 2001 Comparative genomics reveals close genetic relationships between phages from dairy bacteria and pathogenic Streptococci: evolutionary implications for prophage-host interactions Virology 288 325 341 11601904 10.1006/viro.2001.1085

[bib10] Fodor AA Klem ER Gilpin DF Elborn JS Boucher RC Tunney MM 2012 The adult cystic fibrosis airway microbiota is stable over time and infection type, and highly resilient to antibiotic treatment of exacerbations PLoS One 7 e45001 23049765 10.1371/journal.pone.0045001 PMC3458854

[bib11] Fothergill JL Mowat E Ledson MJ Walshaw MJ Winstanley C 2010 Fluctuations in phenotypes and genotypes within populations of *Pseudomonas aeruginosa* in the cystic fibrosis lung during pulmonary exacerbations J Med Microbiol 59 472 481 20019149 10.1099/jmm.0.015875-0

[bib12] Fothergill JL Mowat E Walshaw MJ Ledson MJ James CE Winstanley C 2011 Effect of antibiotic treatment on bacteriophage production by a cystic fibrosis epidemic strain of *Pseudomonas aeruginosa* Antimicrob Agents Chemother 55 426 428 20974862 10.1128/AAC.01257-10 PMC3019632

[bib13] Fothergill JL Walshaw MJ Winstanley C 2012 Transmissible strains of *Pseudomonas aeruginosa* in Cystic Fibrosis lung infections Eur Respir J 40 227 238 22323572 10.1183/09031936.00204411

[bib14] Fothergill JL Ledson MJ Walshaw MJ McNamara PS Southern KW Winstanley C 2013 Comparison of real time diagnostic chemistries to detect *Pseudomonas aeruginosa* in respiratory samples from cystic fibrosis patients J Cyst Fibros 12 675 681 23726365 10.1016/j.jcf.2013.04.007

[bib15] Fothergill JL Neill DR Loman N Winstanley C Kadioglu A 2014 *Pseudomonas aeruginosa* adaptation in the nasopharyngeal reservoir leads to migration and persistence in the lungs Nature Communications 5 4780 10.1038/ncomms5780 25179232

[bib16] Foweraker J 2009 Recent advances in the microbiology of respiratory tract infection in cystic fibrosis Br Med Bull 89 93 110 19155262 10.1093/bmb/ldn050 PMC7109666

[bib17] Ghosh D Roy K Williamson KE Srinivasiah S Wommack KE Radosevich M 2009 Acyl-homoserine lactones can induce virus production in lysogenic bacteria: an alternative paradigm for prophage induction Appl Environ Microbiol 75 7142 7152 19783745 10.1128/AEM.00950-09 PMC2786502

[bib18] Goss CH Burns JL 2007 Exacerbations in cystic fibrosis. 1: epidemiology and pathogenesis Thorax 62 360 367 17387214 10.1136/thx.2006.060889 PMC2092469

[bib19] Hagens S Habel A Blasi U 2006 Augmentation of the antimicrobial efficacy of antibiotics by filamentous phage Microb Drug Resist 12 164 168 17002542 10.1089/mdr.2006.12.164

[bib20] James CE Stanley KN Allison HE Flint HJ Stewart CS Sharp RJ 2001 Lytic and lysogenic infection of diverse *Escherichia coli* and Shigella strains with a verocytotoxigenic bacteriophage Appl Environ Microbiol 67 4335 4337 11526041 10.1128/AEM.67.9.4335-4337.2001 PMC93165

[bib21] James CE Fothergill JL Kalwij H Hall AJ Cottell J Brockhurst MA 2012 Differential infection properties of three inducible prophages from an epidemic strain of *Pseudomonas aeruginosa* BMC Microbiol 12 216 22998633 10.1186/1471-2180-12-216 PMC3544612

[bib22] Kim MS Park EJ Roh SW Bae JW 2011 Diversity and abundance of single-stranded DNA viruses in human feces Appl Environ Microbiol 77 8062 8070 21948823 10.1128/AEM.06331-11 PMC3208976

[bib23] Knezevic P Curcin S Aleksic V Petrusic M Vlaski L 2013 Phage-antibiotic synergism: a possible approach to combatting *Pseudomonas aeruginosa* Res Microbiol 164 55 60 23000091 10.1016/j.resmic.2012.08.008

[bib24] Lawrence JG Hendrix RW Casjens S 2001 Where are the pseudogenes in bacterial genomes Trends Microbiol 9 535 540 11825713 10.1016/s0966-842x(01)02198-9

[bib25] Letkiewicz S Miedzybrodzki R Klak M Jonczyk E Weber-Dabrowska B Gorski A 2010 The perspectives of the application of phage therapy in chronic bacterial prostatitis FEMS Immunol Med Microbiol 60 99 112 20698884 10.1111/j.1574-695X.2010.00723.x

[bib26] Lim YW Evangelista JS 3rd Schmieder R Bailey B Haynes M Furlan M 2014 Clinical insights from metagenomic analysis of sputum samples from patients with cystic fibrosis J Clin Microbiol 52 425 437 24478471 10.1128/JCM.02204-13 PMC3911355

[bib27] Little JW 2005 Lysogeny, prophage induction and lysogenic conversionIn: Waldor MK, Friedman A, Adhya S, (eds)Phages ASM Press: Washington DC 37 54

[bib28] Martin K Baddal B Mustafa N Perry C Underwood A Constantidou C 2013 Clusters of genetically similar isolates of *Pseudomonas aeruginosa* from multiple hospitals in the United Kingdom J Med Microbiol 62 988 1000 23558134 10.1099/jmm.0.054841-0

[bib29] Matsushiro A Sato K Miyamoto H Yamamura T Honda T 1999 Induction of prophages of enterohemorrhagic *Escherichia coli* O157:H7 with norfloxacin J Bacteriol 181 2257 2260 10094706 10.1128/jb.181.7.2257-2260.1999 PMC93641

[bib30] McCallum SJ Corkill J Gallagher M Ledson MJ Hart CA Walshaw MJ 2001 Superinfection with a transmissible strain of *Pseudomonas aeruginosa* in adults with cystic fibrosis chronically colonised by *P aeruginosa* Lancet 358 558 560 11520530 10.1016/s0140-6736(01)05715-4

[bib31] McGrath LT Mallon P Dowey L Silke B McClean E McDonnell M 1999 Oxidative stress during acute respiratory exacerbations in cystic fibrosis Thorax 54 518 523 10335006 10.1136/thx.54.6.518 PMC1745483

[bib32] Mowat E Paterson S Fothergill JL Wright EA Ledson MJ Walshaw MJ 2011 *Pseudomonas aeruginosa* population diversity and turnover in cystic fibrosis chronic infections Am J Respir Crit Care Med 183 1674 1679 21297072 10.1164/rccm.201009-1430OC

[bib33] Ojeniyi B Birch-Andersen A Mansa B Rosdahl VT Hoiby N 1991 Morphology of *Pseudomonas aeruginosa* phages from the sputum of cystic fibrosis patients and from the phage typing set APMIS 99 925 930 1930965

[bib34] Pinheiro JC Bates DM 2000 Linear Mixed-Effects Models: Basic Concepts And Examples Springer: New York

[bib35] Reyes A Semenkovich NP Whiteson K Rohwer F Gordon JI 2012 Going viral: next-generation sequencing applied to phage populations in the human gut Nat Rev Microbiol 10 607 617 22864264 10.1038/nrmicro2853 PMC3596094

[bib36] Wagner PL Acheson DW Waldor MK 2001 Human neutrophils and their products induce Shiga toxin production by enterohemorrhagic *Escherichia coli* Infect Immun 69 1934 1937 11179378 10.1128/IAI.69.3.1934-1937.2001 PMC98107

[bib37] Willner D Furlan M Haynes M Schmieder R Angly FE Silva J 2009 Metagenomic analysis of respiratory tract DNA viral communities in cystic fibrosis and non-cystic fibrosis individuals PLoS One 4 e7370 19816605 10.1371/journal.pone.0007370 PMC2756586

[bib38] Winstanley C Langille MG Fothergill JL Kukavica-Ibrulj I Paradis-Bleau C Sanschagrin F 2009 Newly introduced genomic prophage islands are critical determinants of *in vivo* competitiveness in the Liverpool Epidemic Strain of *Pseudomonas aeruginosa* Genome Res 19 12 23 19047519 10.1101/gr.086082.108 PMC2612960

[bib39] Wright EA Fothergill JL Paterson S Brockhurst MA Winstanley C 2013 Sub-inhibitory concentrations of some antibiotics can drive diversification of Pseudomonas aeruginosa populations in artificial sputum medium BMC Microbiol 13 170 23879797 10.1186/1471-2180-13-170 PMC3726342

