# Supplementary material for: Lytic activity by temperate phages of Pseudomonas aeruginosa in long-term cystic fibrosis chronic lung infections
Source: ISME J. 2014 Dec 2;9(6):1391–8. doi: 10.1038/ismej.2014.223 (PMC4351911; doi:10.1038/ismej.2014.223)
Supplement: Supplementary Data [file ismej2014223x1.doc]

Supplementary data

**Figure S1 Details of patient sputum samples collected over two years**


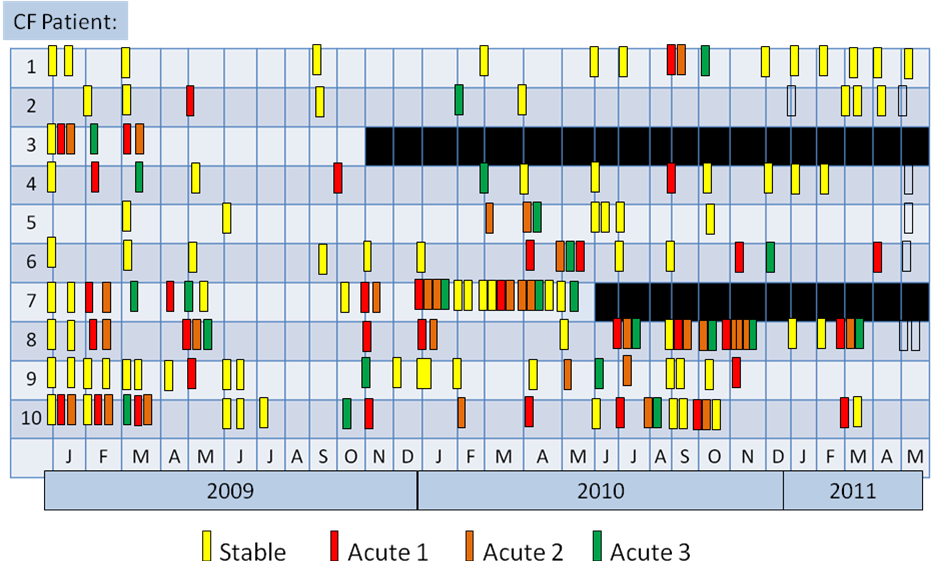


**Figure S1:** CF patient sputum was collected during periods of relative patient health (stable) or exacerbation of symptoms (Acute). Acute 1 samples (red) were taken when patients were first admitted to hospitalised due to exacerbated lung infection. Acute 2 (orange) samples were taken when antibiotic therapy commenced. Acute 3 samples (green) were taken when exacerbations had stabilised and patients were discharged. Full sets of Acute 1, 2 and 3 samples were not always obtained. For the purposes of this study, all acute samples were treated as “exacerbation” and compared with stable samples. Black boxes indicate that the patient died.

Figure S2: Sputasol treatment does not induce LES phages from LESB58 cultures


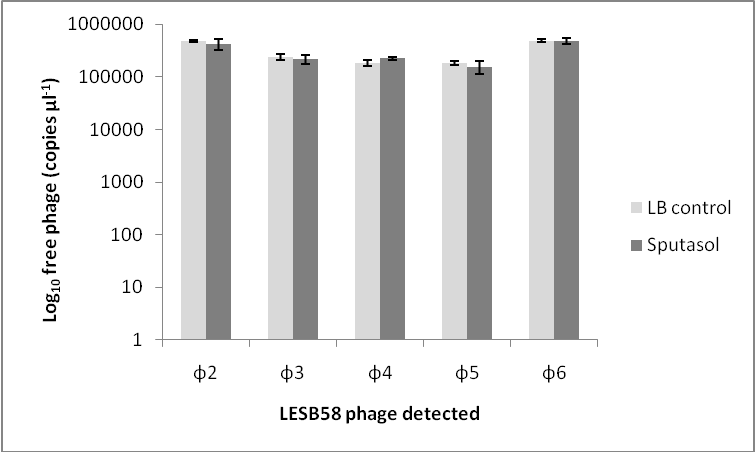


Figure S2: Mid-exponential phase cultures of LESB58 were incubated with equal volumes of Sputasol or LB for 30 minutes, followed by total DNA extraction. Total and prophage copies for each phage were quantified using Q-PCR and used to calculate copies of free phage present. A two-sample t-test comparing free phage production after Sputasol treatment to the LB control was conducted separately for each phage. All results were non-significant at the 0.05 alpha level so a correction for multiple comparisons was not applied (φ2: *t*_(2)_ = 0.53, *p* = 0.65; φ3: *t*_(2)_ = 0.42, *p* = 0.71; φ4: *t*_(2)_ = -1.4, *p* = 0.24; φ5: *t*_(2)_ = 0.72, *p* = 0.55; φ6: *t*_(2)_ = 0.04, *p* = 0.97).

***Table S1:*** ***Primer sequences***

| **Primer** | **Sequence (5′-3′)** | **Target** | **Amplicon (bp)** | **Reference** |
| --- | --- | --- | --- | --- |
| 2*tot*1F | agtagccgacccagaccttt | LESφ2 *int* gene (^*^phage nt 1046-1187) | 141 | James *et al* 2012 |
| 2*tot*1R | atggaagcaaccgagaagtg |
| 2*pro*3F | caagccctgtctggattttc | Between 3’ end of LESφ2 and *tyrS* (PA4139 - PA4138) | 102 | James *et al* 2012 |
| 2*pro*3R | gagacaggttgggagggagt |
| 3*tot*1F | cgcaggtaccaccagacttt | LESφ3 region  (phage nt 2901-3022) | 122 | James *et al* 2012 |
| 3*tot*1R | catgtccagcaggttcaaaa |
| 3*pro*3F | gcggatgttctcaaacgaat | Between 3’ end of LESφ3 and *arsC* (PA3664) | 134 | James *et al* 2012 |
| 3*pro*3R | cgggagaagcaatgacctac |
| 4*tot*1F | gctcatgagtggctgacaac | LESφ4 region  (phage nt 1882-1967) | 105 | James *et al* 2012 |
| 4*tot*1R | tcttgggcagagaaccattc |
| 4*pro*3F | tcgtgctgtgctgatctttt | Between 3’ end of LESφ4 and PA3463 | 172 | James *et al* 2012 |
| 4*pro*3R | agcagtgccagttgatgttg |
| 5*tot*2F | ccaactgggattgtgacaga | LESφ5 *cI* gene  (Phage nt 12477-12581) | 105 | This study |
| 5*tot*2R | ttcaagcaaaacagcagcag |
| 5*pro*5F | ggtttaaggcaacggtcttg | Between 5’ end of LESφ5 and PA2603 | 167 | This study |
| 5*pro*5R | tatgttgcggaaatggtcaa |
| 6*tot*1F | gccctcttggcagtacgata | LESφ6 *int* gene (phage nt 271-398) | 147 | This study |
| 6*tot*1R | aatcaaagcgtgttcgatcc |
| 6*pro*5F | ttgttctgattgaccgaaagg | Between 5’ end of LESφ6 and PA1191 | 146 | This study |
| 6*pro*5R | tgccgagacgtcctagattt |
| gyrPA F1 | cctgaccatccgtcgccacaa | *gyrB* (*P. aeruginosa*) | 221 | Fothergill *et al.* 2013 |
| gyrPA R1 | cgcagcaggatgccgacgc |
| PS21 6 F1 | ttgcaagttggtcaaccgta | PS21 (LES) | 187 | Fothergill *et al.* 2013 |
| PS21 6 R1 | tgaaattccggtttcctttg |

**Table S1: “***tot”* primers amplify unique regions within the phage genome to quantify total phage copies. **“***pro”* primers amplify unique regions that overlap prophage and host sequences to quantify prophage copies*.* The density of Free-phage copies of each LES phage was calculated by subtracting prophage copies from total phage copies in each case. ^*^Phage nucleotide (nt) numbers correspond to prophage sequences reported by .

**Table S2a:** Significance tests for effects on total free phage log density

Variable                   LRT (1 d.f.) P

Time 0.034 0.854

health x bacteria 0.673 0.412

health 1.183 0.277

bacteria 85.9 <0.0001

**Table S2b:** Significance tests for effects on phage-to-bacterium ratio

Variable                   LRT (1 d.f.) P

Bacteria x health 0.126 0.722

Health 0.584 0.445

Time 0.035 0.851

Bacteria 108.4 <0.0001

**Table S2c:** Significance tests for effects on total free phage log density normalised by patient specific mean values (note that explanatory variable bacterial log density was also normalised by patient specific mean values).

Variable LRT (1 d.f.) P

Time 0.0174 0.895

health:bacteria 0.3790 0.374

health 1.290 0.256

bacteria 50.26 <0.0001

**Table S3: Mean free LES phage and host densities for each patient**

| **Patient** | **Mean phage copies µl^-1^ (±SDEV)** | | **Mean host copies µl^-1^ (±SDEV)** | | **Fold difference** |
| --- | --- | --- | --- | --- | --- |
| **CF1** | 4.77E+07 | (± 9.94E+07) | 6.19E+05 | (± 8.05E+05) | 77.06 |
| **CF2** | 3.73E+07 | (± 2.56E+07) | 1.08E+06 | (± 1.72E+06) | 34.54 |
| **CF3** | 7.45E+07 | (± 1.01E+08) | 9.15E+05 | (± 9.00E+05) | 81.42 |
| **CF4** | 2.30E+08 | (± 3.89E+08) | 4.60E+06 | (± 3.41E+06) | 50.00 |
| **CF5** | 7.09E+07 | (± 9.72E+07) | 1.32E+06 | (± 1.71E+06) | 53.71 |
| **CF6** | 1.29E+08 | (± 1.31E+08) | 4.24E+06 | (± 2.96E+06) | 30.42 |
| **CF7** | 1.55E+08 | (± 2.66E+08) | 4.50E+06 | (± 2.78E+06) | 34.44 |
| **CF8** | 1.35E+08 | (± 1.52E+08) | 1.20E+07 | (± 8.75E+06) | 11.25 |
| **CF9** | 3.50E+08 | (± 2.50E+08) | 1.44E+07 | (± 1.18E+07) | 24.31 |
| **CF10** | 1.25E+09 | (± 1.34E+09) | 1.38E+07 | (± 1.37E+07) | 90.58 |

**Table S3:** Free LES phage densities as quantified using Q-PCR. Separate primer sets were used to amplify prophage and total copies of each individual LES phage. Free phage numbers were calculated (total phage copies – prophage copies). Host copies were quantified using ubiquitous *P. aeruginosa* primers.

**Table S4:** **Correlation coefficients between LES phages**

**Table S4:** CF patient sputum contained LES variants that harboured different phage complements. Multivariate correlation analysis of mean free phage densities was used to determine interactions between LES phages (Pearson coeffecients). Black cells indicate absence of prophage. Grey cells indicate intermittent detection of prophage. White cells indicate no significant correlation between free-phage numbers.

| Patients |  | φ 2 | φ 3 | φ 4 | φ 5 | φ 6 |
| --- | --- | --- | --- | --- | --- | --- |
| CF1 (Prophages 3,4, 6) |  |  |  |  |  |  |
|  | φ3 |  |  | 0.49 ( *p = 0.04)* |  |  |
|  | φ4 |  | 0.49 (*p = 0.04)* |  |  |  |
|  |  |  |  |  |  |  |
|  | φ6 |  |  |  |  |  |
| CF2  (Prophages 2,3,4,6) | φ2 |  |  | 0.75 *(p = 0.007)* |  |  |
|  | φ3 |  |  |  |  |  |
|  | φ4 | 0.75 *(p = 0.007)* |  |  |  |  |
|  |  |  |  |  |  |  |
|  | φ6 |  |  |  |  |  |
| CF3  (Prophages 2,3,4,6) | φ2 |  |  | 0.99 *(p = 0.000)* |  |  |
|  | φ3 |  |  |  |  |  |
|  | φ4 | 0.99 (p = 0.000) |  |  |  |  |
|  |  |  |  |  |  |  |
|  | φ6 |  |  |  |  |  |
| CF4  (Prophages 2,3,4,6) | φ2 |  |  | 0.98 *(p = 0.000)* |  |  |
|  | φ3 |  |  |  |  |  |
|  | φ4 | 0.98 *(p = 0.000)* |  |  |  |  |
|  |  |  |  |  |  |  |
|  | φ6 |  |  |  |  |  |
| CF5  (Prophages 2,3,4,6) | φ2 |  |  | 0.95 *(p = 0.000)* |  |  |
|  | φ3 |  |  |  |  |  |
|  | φ4 | 0.95 *(p = 0.000)* |  |  |  |  |
|  |  |  |  |  |  |  |
|  | φ6 |  |  |  |  |  |
| CF6  (Prophages 2,3,4,6) | φ2 |  |  | 0.92 *(p = 0.000)* |  |  |
|  | φ3 |  |  |  |  |  |
|  | φ4 | 0.92 *(p = 0.000)* |  |  |  |  |
|  |  |  |  |  |  |  |
|  | φ6 |  |  |  |  |  |
| CF7  (Prophages 2,3,4 ,*^*^5*, 6) *^*^only in 12/28 samples* | φ2 |  | 0.49 *(p = 0.008)* | 0.99 *(p = 0.000)* |  |  |
|  | φ3 | 0.49 *(p = 0.008)* |  | 0.47 *(p = 0.01)* |  |  |
|  | φ4 | 0.99 *(p = 0.000)* | 0.47 *(p = 0.01)* |  |  |  |
|  | φ5 |  |  |  |  |  |
|  | φ6 |  |  |  |  |  |
| CF8  (Prophages 2,3,4,5,6) | φ2 |  | 0.63 *(p = 0.000)* | 0.85 *(p = 0.000)* | 0.86 *(p = 0.000)* | 0.54 *(p = 0.000)* |
|  | φ3 | 0.63 *(p = 0.000)* |  | 0.35 *(p = 0.045)* | 0.42 *(p = 0.015)* |  |
|  | φ4 | 0.85 *(p = 0.000)* | 0.35 *(p = 0.045)* |  | 0.87 *(p = 0.000)* | 0.51 (*p = 0.002)* |
|  | φ5 | 0.86 *(p = 0.000)* | 0.42 *(p = 0.015)* | 0.87 *(p = 0.000)* |  | 0.41 *(p = 0.019)* |
|  | φ6 | 0.54 *(p = 0.000)* |  | 0.51 (*p = 0.002)* | 0.41 *(p = 0.019)* |  |
| CF9  (Prophages 2,3,4,5,6) | φ2 |  |  | 0.65 *(p = 0.000)* | 0.71 *(p =* *0.000)* |  |
|  | φ3 |  |  |  |  |  |
|  | φ4 | 0.65 *(p = 0.000)* |  |  |  |  |
|  | φ5 | 0.71 *(p =* *0.000)* |  |  |  |  |
|  | φ6 |  |  |  |  |  |
| CF10  (Prophages 2,3,4,*^*^5*,6) *^*^only in 2/27 samples* | φ2 |  |  | 0.90 *(p = 0.000)* |  | 0.52 *(p = 0.005)* |
|  | φ3 |  |  |  |  |  |
|  | φ4 | 0.90 *(p = 0.000)* |  |  |  | 0.65 *(p = 0.000)* |
|  | φ5 |  |  |  |  |  |
|  | φ6 | 0.52 *(p = 0.005)* |  | 0.65 *(p = 0.000)* |  |  |
